# Expression of Transient Receptor Potential Ankyrin 1 (TRPA1) and Its Role in Insulin Release from Rat Pancreatic Beta Cells

**DOI:** 10.1371/journal.pone.0038005

**Published:** 2012-05-31

**Authors:** De-Shou Cao, Linlin Zhong, Tsung-han Hsieh, Mruvil Abooj, Mahendra Bishnoi, Lauren Hughes, Louis S. Premkumar

**Affiliations:** Department of Pharmacology, Southern Illinois University School of Medicine, Springfield, Illinois, United States of America; Indiana University School of Medicine, United States of America

## Abstract

**Objective:**

Several transient receptor potential (TRP) channels are expressed in pancreatic beta cells and have been proposed to be involved in insulin secretion. However, the endogenous ligands for these channels are far from clear. Here, we demonstrate the expression of the transient receptor potential ankyrin 1 (TRPA1) ion channel in the pancreatic beta cells and its role in insulin release. TRPA1 is an attractive candidate for inducing insulin release because it is calcium permeable and is activated by molecules that are produced during oxidative glycolysis.

**Methods:**

Immunohistochemistry, RT-PCR, and Western blot techniques were used to determine the expression of TRPA1 channel. Ca^2+^ fluorescence imaging and electrophysiology (voltage- and current-clamp) techniques were used to study the channel properties. TRPA1-mediated insulin release was determined using ELISA.

**Results:**

TRPA1 is abundantly expressed in a rat pancreatic beta cell line and freshly isolated rat pancreatic beta cells, but not in pancreatic alpha cells. Activation of TRPA1 by allyl isothiocyanate (AITC), hydrogen peroxide (H_2_O_2_), 4-hydroxynonenal (4-HNE), and cyclopentenone prostaglandins (PGJ_2_) and a novel agonist methylglyoxal (MG) induces membrane current, depolarization, and Ca^2+^ influx leading to generation of action potentials in a pancreatic beta cell line and primary cultured pancreatic beta cells. Activation of TRPA1 by agonists stimulates insulin release in pancreatic beta cells that can be inhibited by TRPA1 antagonists such as HC030031 or AP-18 and by RNA interference. TRPA1-mediated insulin release is also observed in conditions of voltage-gated Na^+^ and Ca^2+^ channel blockade as well as ATP sensitive potassium (K_ATP_) channel activation.

**Conclusions:**

We propose that endogenous and exogenous ligands of TRPA1 cause Ca^2+^ influx and induce basal insulin release and that TRPA1-mediated depolarization acts synergistically with K_ATP_ channel blockade to facilitate insulin release.

## Introduction

The Transient Receptor Potential (TRP) channels TRPC (canonical), TRPV (vanilloid), TRPM (melastatin), TRPP (polycystin), TRPML (mucolipin), and TRPA (ankyrin) are involved in diverse functions that include transduction of sensory information, cell growth/death, and neurotransmitter/hormone release [1]. Nociceptive TRP channels expressing C-fibers have been shown to innervate the pancreas and are involved in carrying pain sensation and glucose homeostasis. Proinflammatory agents that activate/sensitize TRP channels can cause neurogenic inflammation and pain. Nociceptors express these TRP channels and mediate the excruciating pain associated with pancreatitis [2]–[4]. However, it is becoming evident that several of these TRP channels are expressed in nonsensory tissues and are involved in functions other than somatic sensation, which include their expression in pancreatic beta cells and involvement in insulin release. TRPV1 has been shown to modulate insulin secretion in rat pancreatic beta cells [5]. Both TRPV1 and TRPA1 are functionally expressed in pancreatic INS-1 beta cells [6] and RINm5F cells [7]. TRPV2-mediated insulin release by high glucose stimuli is significantly reduced after TRPV2 knockdown [8]. Amyloid polypeptide-induced an increase in intracellular Ca^2+^ in pancreatic beta cells is mediated by TRPV4 [9]. TRPM2 is highly expressed in pancreatic beta cells and regulates insulin release [10], [11]. Activation of TRPM3 channel by the neurosteroid pregnenolone sulphate triggers Ca^2+^ influx and promotes insulin release [12]. TRPM4 has been shown to control insulin secretion in pancreatic beta cells [13], [14], and recently, a role for TRPM5 was indicated by the finding of altered Ca^2+^ oscillations in beta cells from TRPM5 knockout animals [15]. Although these TRP channels are expressed in pancreatic beta cells and are associated with insulin release, the efficacy and the endogenous ligands for these channels are far from clear.

TRPA1 is a Ca^2+^ permeable nonselective cation channel, which is expressed in trigeminal and dorsal root ganglion neurons [16]. TRPA1 has been shown to be activated by several reactive electrophilic food ingredients and irritants like allyl isothiocyanate (AITC), cinnamaldehyde, allicin and acrolein, but also by non-reactive sensory compounds like methylsalicylate and icilin [17]–[21]. It has been proposed that TRPA1 is involved in various sensory processes, such as the detection of noxious cold, mechanosensation, and inflammatory hyperalgesia [16], [17], [22]–[26]. TRPA1 is an attractive candidate to be involved in insulin release because it can be activated by molecules that are produced during oxidative phosphorylation such as hydrogen peroxide (H_2_O_2_) and 4-Hydroxynonenal (4-HNE) and cyclopentenone prostaglandins (PGJ_2_) [27], [28]. Methylglyoxal (MG) is formed from triose phosphates during secondary glucose metabolism in hyperglycemic condition. It is well known that MG covalently modifies arginine, lysine and cysteine residues and forms advanced glycation end products, leading to hyperglycemia-induced tissue damage [29]–[31]. It has been reported that MG has been shown to cause membrane depolarization and insulin release in rat pancreatic beta cells [32], [33]. Given the finding that TRPA1 can be activated by covalent modifying agents [34], [35], we propose that MG can be a potential agonist for TRPA1.

Pancreatic beta cells in the islets of Langerhans release insulin in response to increases in blood glucose levels. This involves the production of adenosine triphosphate (ATP) that blocks ATP-sensitive potassium (K_ATP_) channels, which depolarizes beta cells and promotes Ca^2+^ influx [36]. During glucose metabolism, reactive oxygen species (ROS) and MG are produced as byproducts [27], [37]–[39]. We hypothesize that activation of TRPA1 by these metabolites could play a role in insulin release from pancreatic beta cells. Further, a number of studies have proposed that there is a K_ATP_ channel-independent pathway of insulin release in pancreatic beta cells. Since this pathway is also activated by glucose metabolism, it is considered to be an amplifying pathway. But the mechanisms underlying this amplification process are not fully understood [40]–[42].

In this study, we have characterized the expression and function of TRPA1 channels in pancreatic beta cells using a rat beta cell line (RINm5F cells) and freshly isolated rat pancreatic islets. We show utilizing a variety of techniques that activation of TRPA1 by endogenous and exogenous ligands causes membrane depolarization, Ca^2+^ influx and insulin release.

## Methods

### Isolation of rat pancreatic islets and beta cells

All procedures used in this study were approved by the animal care and use committee at Southern Illinois University, School of Medicine, and conformed according to National Institutes of Health and institutional guidelines. Sprague Dawley rats (Harlan laboratories, Indianapolis, IN, USA) were anesthetized using intraperitoneal injection of ketamine (85 mg/kg) and xylazine (10 mg/kg). The common bile duct was clamped and cannulated with a blunt needle. The pancreas was distended with 5 ml of cold Hanks' balanced salt solution containing collagenase 1 mg/ml (Roche chemicals, Indianapolis, IN, USA) and 7.5 mM CaCl_2_ with pH 7.8. The pancreas was removed and incubated for 15 min in a water bath at 38°C. After centrifugation, the pellet was washed twice and filtered through a mesh filter. Following centrifugation, the pellet was resuspended in 1.080 g/ml optiprep and layered under a discontinuous optiprep gradient (1.075, 1.070, and 1.060) (Sigma Aldrich, St Louis, MO, USA). After centrifugation, the tissue at the 1.070/1.060 interface was collected and cultured in Rosewell Park Memorial Institute (RPMI) 1640 medium (Invitrogen, Carlsbad, California, USA) supplemented with 10% FBS and antibiotics. In some of the experiments individual islets were used. For other studies, islets were digested into individual cells by incubation with trypsin and plated on the cover slips.

### Cell culture and transfection

For cell culture studies, human embryonic kidney (HEK) 293T cells (Clontech Laboratories, Inc, Palo Alto, CA, USA) were cultured in DMEM with 10% fetal bovine serum (FBS) and penicillin (50 units/ml)-streptomycin (25 µg/ml) (Gibco-Invitrogen, Carlsbad, California, USA). TRPA1 cDNA and GFP cDNA were co-transfected into HEK 293T cells with Lipofectamine 2000 reagent following manufacture's protocol (Invitrogen, Carlsbad, California, USA). The fluorescent cells were used for recording currents 24 hrs after transfection. The non-fluorescent cells were used as a negative control. RINm5F cells (rat pancreatic beta cell line) and INR1G9 cells (hamster glucagonoma alpha cell line) were gift from Henry Cheng, Louisiana State University, Baton Rouge, LA, and grown in medium supplemented with 10% FBS.

### Immunohistochemistry and peptide absorption studies

Rats were anesthetized using intraperitoneal injection of ketamine (85 mg/kg, i.p.) and xylazine (10 mg/kg, i.p.) and perfused transcardially with 4% paraformaldehyde. The pancreas was harvested and quickly frozen. The pancreas was cut into 30 µm sections using a microtome (Leica CM 1850, North Central Instruments Inc, Plymouth, MN, USA). The sections were rinsed in 0.1 M PBS, and permeabilized with 1% triton X-100 in PBS for 30 minutes. The sections were then blocked in 10% normal donkey serum in PBS for 30 minutes. The sections were incubated with rabbit anti-TRPA1 antibody (1∶100, Osenses, Australia) and mouse anti-insulin antibody (1∶200, Millipore, Billerica, MA, USA) overnight at 4°C. Rhodamine donkey anti-mouse IgG (1∶ 100, Jackson Immunoresearch Laboratories Inc., West Grove, PA, USA) and FITC donkey anti-rabbit/guinea pig IgG (1∶ 100, Jackson Immunoresearch) were used. Images were captured by a fluorescence microscope.

For peptide absorption studies, TRPA1 blocking peptide (1∶50, Osenses, Australia) that was used to generate the antibody was incubated with TRPA1 antibody for 1 hr at room temperature before the experiment. The sections were incubated with the mixture of TRPA1 peptide and rabbit anti-TRPA1 antibody (1∶100, Osenses, Australia) and mouse anti-insulin antibody (1∶200, Millipore, Billerica, MA, USA) overnight at 4°C. Rhodamine donkey anti-mouse IgG (1∶ 100, Jackson Immunoresearch Laboratories Inc., West Grove, PA, USA) and FITC donkey anti-rabbit/guinea pig IgG (1∶100, Jackson Immunoresearch) were used. Images were captured by a fluorescence microscope.

### Total RNA extraction and RT-PCR

Total RNA was extracted by Trizol reagent (Invitrogen Co., Carlsbad, California, USA) from rat dorsal root ganglia (DRG), rat islets, rat pancreas, RINm5F cells and INR1G9 cells and cDNAs prepared by reversely transcriptioned to cDNAs using a cDNA sythesis kit (Promega Corporation, Madison, WI, USA). PCR was performed by using different cDNAs as templates in 35 cycles with 30 s denaturation at 95°C, 30 s annealing at 58°C, and 30 s extension at 68°C by using PCR green master mix (Promega Corporation, Madison, WI, USA). The PCR primers for rats were as follows: TRPA1, F: TGCCCTTATTCCTCGACATC, R: CAGTTCCACCTGCATAGCAA; β-actin, F: AGCCATGTACGTAGCCATCC, R: AGGAAGGAAGGCTGGAAGAG. The PCR products were electrophoresed in 1.5% agarose gel with ethidium bromide in TBE buffer. The gel was scanned by Versa Doc imaging system (Bio-Rad, Hercules, CA, USA) and the blot band density was quantified by Quantity One (Bio-Rad).

### Western blotting

RINm5F cells were collected in a lysis buffer (0.1% SDS, 1% Triton X-100, 1% deoxycholate, protease and phosphatase inhibitor cocktail, 1∶100, Sigma Aldrich, St Louis, MO, USA), homogenized and centrifuged. The protein concentration was measured by the bicinchoninic acid (BCA) assay. Proteins were separated by 10% SDS-PAGE and transferred to a nitrocellulose membrane (Bio-Rad, Hercules, CA). Membranes were probed overnight with rabbit anti-TRPA1 (1∶500, Osenses, Australia) and β-actin (1∶200, Sigma Aldrich, St Louis, MO, USA) antibodies followed by incubation with horseradish peroxidase-conjugated (HRP) goat anti-rabbit IgG (1∶10,000, Santa Cruz biotechnology Inc., Santa Cruz, CA, USA) for 1 hr. After incubation with enhanced chemiluminescence reagents (Santa Cruz biotechnology Inc.), membranes were analyzed using a Hitachi Genetic Systems (Hitachi Software Engineering, Japan).

### Ca^2+^ fluorescence imaging

RINm5F cells and cultured primary pancreatic beta cells grown on glass cover slips were incubated with 3 µM Fluo-4 AM (Invitrogen) for 30 min at 37°C and washed with physiological buffer containing the following (in mM): 140 NaCl, 10 HEPES, 2 CaCl_2_, 1.2 MgCl_2_, 5 KCl, 5.5 glucose, pH 7.35. Fluo-4 was excited at 488 nm, and emitted fluorescence was filtered with a 535±25 nm band pass filter. The ratio of the fluorescence change F/F_o_ was plotted to represent the change in intracellular Ca^2+^ levels.

### Whole-cell patch-clamp recording

RINm5F cells and cultured primary pancreatic beta cells grown on poly-D-lysine-coated cover slips were used for recording TRPA1 currents. For whole-cell patch-clamp recordings, the bath solution contained (in mM): 140 Na gluconate, 5 KCl, 10 HEPES, 1 MgCl_2_, 1.5 EGTA/2 CaCl_2_, pH adjusted to 7.35 with NaOH and the pipette solution contained (in mM): 140 K gluconate, 5 KCl, 10 HEPES, 2 MgCl_2_, 10 EGTA, 2 K_2_ATP, 0.5 GTP, pH adjusted to 7.35 with KOH. Currents were recorded with a holding potential at −60 mV using an Axopatch 200B integrating patch-clamp amplifier (Axon Instruments Inc.). Membrane potentials and action potentials were recorded in the current-clamp mode. All experiments were conducted at room temperature. Data were digitized (VR-10B; InstruTech, Great Neck, NY) and stored on a computer using a LabView interface (National Instruments). For analysis, data were filtered at 2.5 kHz (−3 dB frequency with an eight-pole low-pass Bessel filter; LPF-8; Warner Instruments) and digitized at 5 kHz. Current amplitudes and membrane potentials were measured using Channel 2 (software kindly provided by Michael Smith, Australian National University, and Canberra, Australia).

### Insulin release assays

Insulin release studies were performed in both RINm5F cells and isolated pancreatic islets. RINm5F cells were cultured in 12-well plates for 24 hrs and used for measuring insulin release. After equilibration with Kreb-Ringer (KR) solution containing (in mM): NaCl 136, KCl 4.8, CaCl_2_ 2.5, KH_2_PO4 1.2, MgCl_2_ 1.2, NaHCO_3_ 5, HEPES 10 and 0.05% BSA, the cells were incubated with AITC, MG, 4-HNE, PGJ_2_ and HC 030031 for 1 hr in 0.4 ml KR solution and the solution was collected. The insulin content was measured by using ultra sensitive rat insulin ELISA kits (Crystal Chem Inc. Downers Grove, IL, USA) according to the manufacturer's protocol. For insulin release studies in isolated islets, they were cultured overnight in RPMI 1640 medium. Five islets in each group were hand-picked and incubated with or without treated reagents in 0.5 ml KR solution at 37°C for 15 min. Then the supernatants were collected for ELISA assay using ultra sensitive rat insulin ELISA kits (Crystal Chem Inc. Downers Grove, IL, USA).

### Short interference RNA (siRNA) knockdown of TRPA1

siRNA sequences were designed from the rat TRPA1 sequence and synthesized by Ambion (Austin, TX, USA). The siRNAs against rat TRPA1 were as follows: F: GGUCCAACAUAACCGCAUATT; R: UAUGCGGUUAUGUUGGACCAT and negative control siRNA provided by the manufacturer (proprietary and the sequence was not disclosed) were used as mock transfected (Ambion Inc., Austin, TX, USA). The siRNAs were transfected using lipofectamine 2000 reagent (Invitrogen Co.) following the manufacturer's protocol. The cells were used 2–3 days after transfection.

### Data analysis

Data are represented as mean ± S.E.M. (Standard Error of the Mean). Student's t-test and one way analysis of variance (ANOVA) were used for statistical comparisons and the significance is considered at *P*<0.05.

## Results

### Expression of TRPA1 in pancreatic beta cells

**Figure 1:**
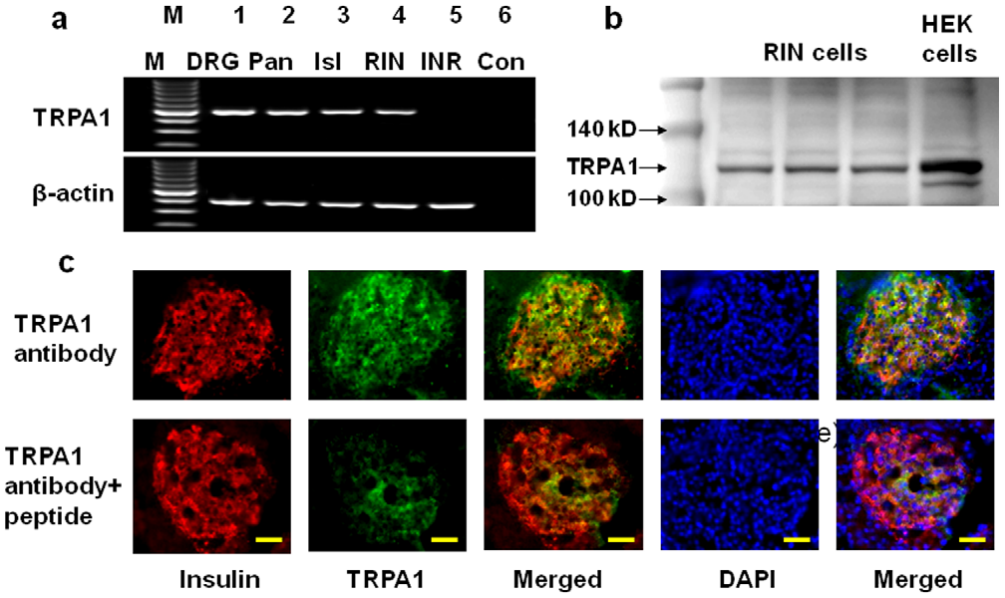
Expression of TRPA1 in pancreatic beta cells. **a.** RT-PCR shows the expression of TRPA1 mRNA in DRG neurons, whole pancreas (Pan), isolated islets (Isl), a pancreatic beta cell line (RIN), but not in a pancreatic alpha cell line (INR). **b.** Western blots show the expression of TRPA1 protein in RIN cells and HEK cells heterologously expressing TRPA1. c. Immunostaining of insulin (red), TRPA1 (green), and the co-expression (yellow) in the pancreatic islet (top panel). When the slices were incubated with the TRPA1 antibody after preabsorbing with a peptide used for making the antibody, the TRPA1 staining was considerably reduced (lower panel). The nuclei were stained with DAPI (scale bar=100 µM).

Using RT-PCR technique, the presence of TRPA1 channel was detected in dorsal root ganglion (DRG), pancreas (Pan) and pancreatic islets (Isl), and in rat pancreatic beta cell line RINm5F (RIN), but not in a hamster pancreatic alpha cell line, INR1G9 (INR) (Fig. 1a). TRPA1 protein was detected in RIN cells using Western blot technique (Fig. 1b). In addition, immunohistochemical studies revealed that TRPA1 was abundantly and selectively expressed in pancreatic islets and TRPA1-positive cells were co-stained with insulin in rat pancreatic islets (Fig. 1c). The specificity of antibody binding was confirmed by preabsorbing the antibody by incubating with a TRPA1 blocking peptide (Fig. 1c).

### Functional characterization of TRPA1 in pancreatic beta cells by Ca^2+^ fluorescent imaging

**Figure 2:**
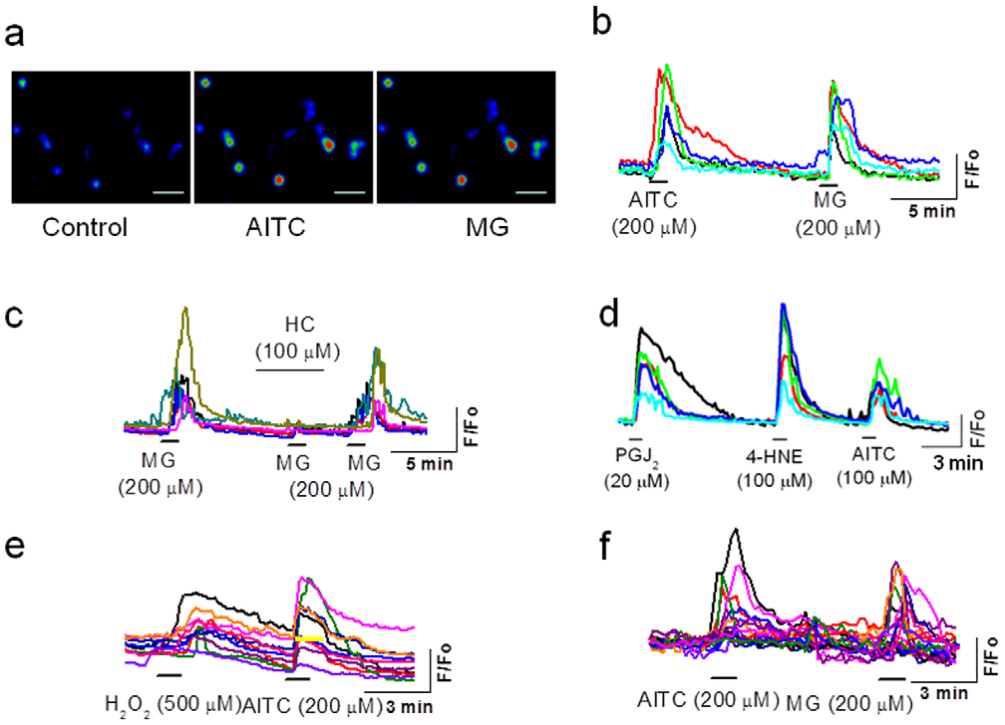
TRPA1-mediated Ca^2+^ **influx in pancreatic beta cells.** **a, b.** Application of AITC and MG induce an increase in intracellular Ca^2+^ in RIN cells (size of the bar is 100 µM). **c.** MG-induced Ca^2+^ influx is inhibited by TRPA1 antagonist HC030031. **d.** Ca^2+^ influx induced by endogenous ligands PGJ_2_, 4-HNE, and AITC in RIN cells. **e.** Ca^2+^ influx induced by H_2_O_2_ and AITC in RIN cells. **f.** AITC-and MG-induced an increase in intracellular Ca^2+^ in rat cultured primary pancreatic beta cells.

We characterized the TRPA1 channel function in pancreatic beta cells using Ca^2+^ fluorescence imaging. Exposure of RIN cells to the exogenous TRPA1 agonist allylisothiocyanate (AITC, 200 µM) induced a significant increase in intracellular Ca^2+^ levels (similar in magnitude to those observed in DRG neurons (fold change, beta cells, 1.38±0.03, n=28; DRG neurons 1.41±0.03, n=26) (Fig. 2a, b). In an effort to identify novel endogenous ligands, we used the dicarbonyl compound methylglyoxal (MG), which is produced during glucose metabolism and in higher levels in hyperglycemic conditions. MG (200 µM) induced a significant increase in intracellular Ca^2+^ levels (fold change 1.35±0.03, n=28) in RIN cells (Fig. 2a, b). In the presence of a TRPA1 antagonist, HC 030031 (100 µM), MG-induced elevation of intracellular Ca^2+^ levels was completely blocked (Fig. 2c). MG (1–1000 µM) dose dependently increased Ca^2+^ influx in RIN cells with an EC_50_ value of 28.6 µM (Fig. S1). Other TRPA1 agonists such as PGJ_2_ (20 µM), 4-HNE (100 µM) and H_2_O_2_ (500 µM), induced significant Ca^2+^ influx in RIN cells (fold change, H_2_O_2_, 1.21±0.02, n=23; 4-HNE, 1.37±0.04, n=27; PGJ_2_, 1.32±0.03, n=20) (Fig. 2d, e). AITC (200 µM) and MG (200 µM) also induced Ca^2+^ influx in freshly isolated primary beta cells from rats (fold change, AITC, 1.17±0.01; MG, 1.19±0.03, n=17) (Fig. 2f). In the absence of extracellular Ca^2+^, application of TRPA1-agonists did not induce an increase in intracellular Ca^2+^ levels, thus ruling out the possibility of Ca^2+^ release from an intracellular source. In similar experimental conditions, application of AITC, MG, 4-HNE, PGJ_2_ or H_2_O_2_ did not increase intracellular Ca^2+^ levels in a pancreatic alpha cell line (data not shown).

### Functional characterization of TRPA1 in pancreatic beta cells by electrophysiological techniques

**Figure 3:**
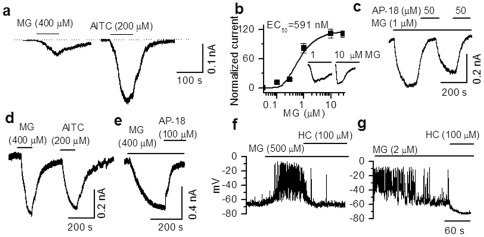
TRPA1-mediated membrane currents in primary pancreatic beta cells. **a.** Membrane currents induced by extracellular application of MG and AITC in primary pancreatic beta cells. **b.** A concentration-response curve of membrane currents induced by MG included in the pipette solution in primary beta cells (EC_50_=0.59 µM). Lower concentrations (∼1 µM) of MG are sufficient to induce maximal currents when applied intracellulary (inset), but the time to peak with lower concentrations is longer and the desensitization is profound at higher concentrations. **c.** Currents evoked by intracellular application of MG are reversibly blocked by extracellular application of AP-18. **d.** Currents elicited by MG and AITC in HEK 293T cells heterologously expressing TRPA1. **e.** MG-induced currents can be blocked by AP-18. **f.** Under current clamp conditions, extracellular application of MG depolarizes the membrane and generates action potentials that could be blocked by HC030031. **g.** Intracellular application of MG causes a robust depolarization and generates action potentials that could be blocked by HC030031.

We used voltage-clamp technique and recorded TRPA1-mediated currents from primary rat pancreatic beta cells and RIN cells. In primary rat pancreatic beta cells, significant membrane currents were induced by application of MG (400 µM) (10.8±1.3 pA/pF, range 6.0–25.1 pA/pF, n=15) and AITC (200 µM) (17.2±2.9 pA/pF, range 7.1–33 pA/pF, n=18) (Fig. 3a). Relatively high concentrations (>100 µM) of MG were required to elicit currents or cause Ca^2+^ influx. The concentration of MG in the plasma has been estimated to be between 1–2 µM in normal individuals and 2–5 µM in hyperglycemic conditions [43]. However, the cytoplasmic concentration of MG is not known. We reasoned that being a reactive molecule generated intracellularly, MG may not cross the plasma membrane readily. Therefore, we exposed the intracellular surface of the membrane to MG by including different concentrations of MG (0.03 to 10 µM) in the pipette solution. The current recordings were started as soon as the whole-cell configuration was achieved by applying negative pressure. In this condition, MG-induced currents in the sub-micromolar range. The EC_50_ value was found to be 0.59 µM (Fig. 3b). Interestingly, currents induced by 1 and 10 µM MG differed in the activation phase (10–90% rise time: 1 µM 134±13.9 s, n=3; 10 µM, 80±6.8 s, n=3), but almost had similar amplitudes in a few experiments (1 µM, 8.18±0.98 pA/pF, n=4; 10 µM, 11.18±0.97 pA/pF, n=3) (Fig. 3b, inset) suggesting that even lower concentrations of MG could induce maximal responses over time. Intracellular MG-induced current was blocked completely by TRPA1 antagonist (AP-18, 50 µM) applied extracellularly (Fig. 3c). Similarly, in RIN cells the TRPA1 agonists AITC, MG, 4-HNE, and PGJ_2_ induced currents, that were blocked by HC030031 (100 µM) (Fig. S2). In order to compare the characteristics of TRPA1-mediated currents in the pancreatic beta cells, we recorded currents from HEK293T cells heterologously expressing TRPA1. MG (400 µM) induced currents (15.0±0.6 pA/pF, n=13) of similar magnitude to that induced by AITC (200 µM) (19.1±0.7 pA/pF, n=10) (Fig. 3d). As demonstrated in pancreatic beta cells, TRPA1 agonist evoked currents in HEK cells were completely blocked by a selective TRPA1 antagonist (AP-18, 100 µM) (Fig. 3e).

Next, using the current-clamp technique, we showed that application of AITC (20 µM) and MG (500 µM) induced membrane depolarizations (AITC, 26.1±1.9 mV, n=9; MG, 24.1±3.1 mV, n=11) and increased the frequency of action potentials (AITC, 0.17±0.11 to 0.87±0.14 Hz, n=6; MG, 0.04±0.04 to 1.12±0.28 Hz, n=4) in primary pancreatic beta cells, which could be blocked by HC030031 (100 µM) (AITC, 3.38±1.97 mV, n=5; MG, 1.26±2.61 mV, n=6). Figure 3f shows that extracellular application of MG (500 µM) induced a depolarization that could be blocked by HC030031 (100 µM). As shown in voltage-clamp experiments, when a lower concentration of MG (2 µM) was included in the pipette solution, it induced significant membrane depolarization (21.5±2.5 mV, n=7) and increased the frequency of action potentials (0.08±0.04 to 1.14±0.24 Hz, n=5). This effect could also be blocked by HC030031 (100 µM) applied extracellularly (1.26±2.61 mV, n=6) (Fig. 3g).

### TRPA1-mediated insulin release in pancreatic beta cells

**Figure 4:**
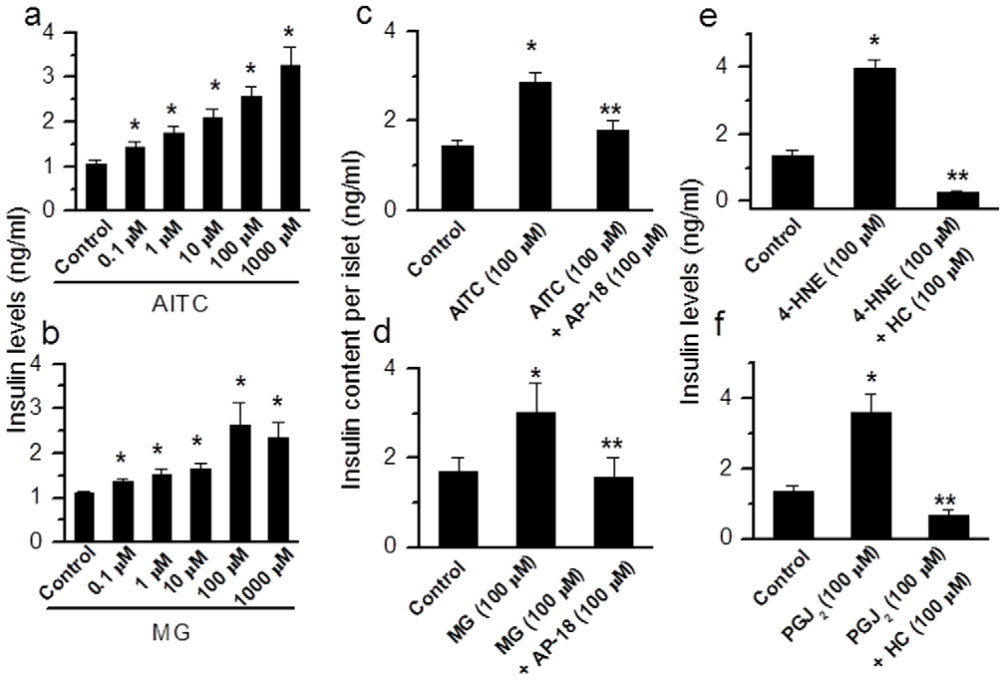
TRPA1-mediated insulin release in pancreatic beta cell line and primary isolated pancreatic islets. **a,b.** Dose-dependent increase in insulin release induced by AITC (0.1–1000 µM, **n=7**) and MG (0.1–1000 µM, **n=5**) in RIN cells (* p<0.05). **c,d.** AITC and MG induce a significant increase (AITC, n=11,* p<0.001; MG, n=10 * p=0.004) in insulin release from primary isolated pancreatic beta cell islets that could be blocked by the specific TRPA1 antagonist AP-18 (AITC+AP-18, n=6, ** p<0.001; MG+AP-18, n=6, ** p=0.008). **e.** 4-HNE (100 µM)-induced insulin release is inhibited by HC030031 (100 µM) (4-HNE, n=6, * p<0.001; 4-HNE+HC030031, n=3, ** p<0.001). **f.** PGJ_2_ (20 µM)-induced insulin release is inhibited by HC030031 (100 µM) (PGJ_2_, n=6, * p<0.001; PGJ_2_+HC030031, n=3, ** p<0.001).

In order to determine the physiological relevance of TRPA1 expressed in pancreatic beta cells, we investigated the ability of TRPA1 agonists to cause insulin release in RIN cells and isolated intact islets from rat pancreas. In RIN cells, application of AITC (0.1–1000 µM) and MG (0.1–1000 µM) caused a dose-dependent increase in insulin release (Fig. 4a, b). Since we do not know the intracellular concentrations of MG achieved, the EC_50_ value obtained by extracellular application is only used to compare the relative potency between agonists. In intact islets, AITC (100 µM) (basal 1.45±0.1; AITC, 2.88±0.2 ng/ml, n=11, p<0.001 and MG (100 µM) (basal 1.69±0.32, MG, 3.00±0.69 ng/ml, n=10, p<0.01) induced significant insulin release that could be blocked by incubation with AP-18 (100 µM) (AITC+AP-18, 1.79±0.20 ng/ml, n=6, p<0.001; MG+AP-18, 1.58±0.42 ng/ml, n=6, p<0.01) (Fig. 4c.d). We also determined insulin release caused by other TRPA1 agonists. In RIN cells 4-HNE (100 µM) 3.96±0.28 ng/ml, n=6, p<0.001 and PGJ_2_ (100 µM) 3.59±0.51 ng/ml, n=6, p<0.001) induced significant increases in insulin release that were inhibited by HC030031 (100 µM) (4-HNE+HC, 0.28±0.05 ng/ml, n=3, p<0.001; PGJ_2_+HC, 0.66±0.17 ng/ml, n=3, p<0.001) (Fig. 4 e, f).

**Figure 5:**
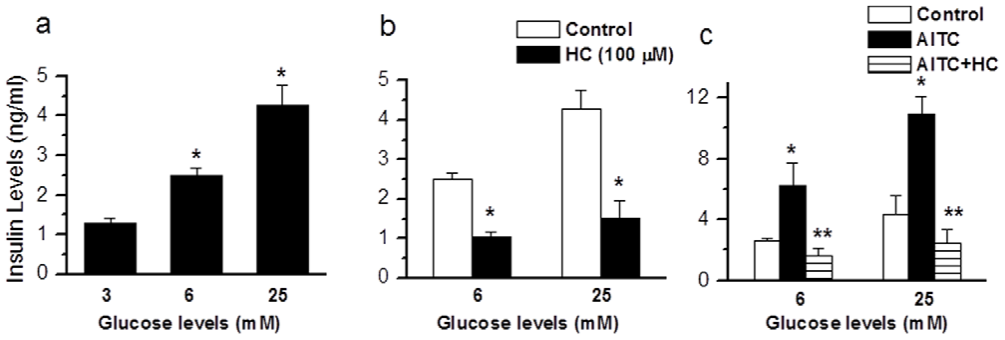
Insulin release induced by different concentrations of glucose. a. Insulin release induced by different concentrations of glucose (6 mM, n=8, * p<0.001; 25 mM, n=9, *p<0.001) **b.** Insulin release induced by different concentrations of glucose is inhibited by HC030031 (100 µM) (6 mM, n=4, * p<0.001; 25 mM, n=7, * p<0.001, as compared to control). **c.** Insulin release induced by AITC (200 µM) in different concentrations of glucose is inhibited by HC030031 (100 µM) (6 mM, AITC, n=4, * p<0.01, AITC+HC030031, n=4, ** p<0.001; 25 mM, AITC, n=4, * p=0.023, AITC+HC030031, n=4, ** p<0.01).

In order to examine the role of TRPA1 in glucose-induced insulin release, we determined insulin release in response to incubation of cells with glucose (6 and 25 mM) and tested the effect of a TRPA1 antagonist. A dose-dependent increase in insulin release was observed with increasing concentrations of glucose (6 mM, increased from 1.28±0.12 to 2.50±0.16 ng/ml, n=8, p<0.001; 25 mM, 4.27±0.48 ng/ml, n=9, p<0.001) (Fig. 5a). Glucose-induced insulin release could be partially inhibited by HC030031 (100 µM) (6 mM, decreased from 2.52±0.16 to 1.05±0.12 ng/ml, n=4, p<0.001; 25 mM, decreased from 4.28±0.48 to 1.52±0.44 ng/ml, n=7 p<0.001) (Fig. 5b), suggesting that TRPA1 plays a role in glucose-induced insulin release. Further, AITC-induced insulin release at different concentrations of glucose (6 mM, increased from 2.62±0.14 to 6.25±1.46 ng/ml, n=4, p<0.01; 25 mM, increased from 4.37±1.17 to 10.93±1.18 ng/ml, n=4, p<0.05) could be significantly inhibited by HC030031 (100 µM) (6 mM, 1.64±0.48 ng/ml, n=4, p<0.001; 25 mM, 2.47±0.88 ng/ml, n=7 p<0.001) (Fig. 5c).

### TRPA1-mediated insulin release is independent of voltage-gated Na^+^ and Ca^2+^ channels or K_ATP_ channels

**Figure 6:**
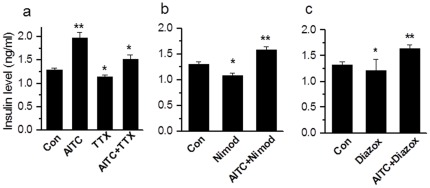
TRPA1-mediated insulin release is independent of voltage-gated Na^+^, Ca^2+^ and K_ATP_ channels. **a.** AITC caused a significant increase in insulin release (n=6, ** p<0.01). The basal insulin release is inhibited by incubation of RIN cells with TTX (1 µM) (TTX, n=6, * p<0.05. When challenged with AITC (200 µM), there is a significant increase in insulin release AITC+TTX, n=6, * p<0.05 as compared to TTX. **b.** In the presence of Ca^2+^ channel blocker nimodipine (5 µM) basal insulin release is decreased significantly (n=6, * p<0.05), but there is a significant increase when challenged with AITC+nimodipine (n=6,** p<0.01). **c.** In the presence of K_ATP_ channel opener, diazoxide (200 µM), basal insulin release is significantly decreased (n=6, * p<0.05), when challenged with AITC, there is a significant increase in insulin release (n=3, ** p<0.01).

In order to address whether Ca^2+^ influx caused by TRPA1 activation is sufficient to induce insulin release or whether TRPA1-induced membrane depolarization and Ca^2+^ influx through voltage-gated Na^+^ and Ca^2+^ channels are required, we used tetrodotoxin (TTX) to block voltage-dependent Na^+^ channels and nimodipine to block voltage-gated Ca^2+^ channels in RIN cells. AITC significantly increased insulin levels (from 1.29±0.04 to 1.97±0.12 ng/ml). In the presence of TTX (1 µM), insulin release was significantly inhibited (1.15±0.03 ng/ml, n=6, p<0.01), however, when stimulated by AITC (100 µM) in the presence of TTX, insulin release was significantly increased (1.52±0.09 ng/ml, n=6, p<0.05) suggesting a direct role of TRPA1-mediated Ca^2+^ flux in insulin release (Fig. 6a). In the presence of voltage-gated Ca^2+^ channel blocker nimodipine (5 µM), the basal insulin release was significantly reduced (from 1.31±0.05 to 1.09±0.04 ng/ml, n=6, p<0.05), but following administration of AITC in the presence of nimodipine, a significant increase in insulin release was observed (1.58±0.07 ng/ml, n=6, p<0.01) suggesting that activation of TRPA1 can induce insulin release independent of voltage-gated Ca^2+^ channels. Then, we used diazoxide (200 µM) to activate K_ATP_ channels and keep the membrane potential hyperpolarized. In this condition, a significant decrease in basal insulin release occurred (from 1.32±0.06 to 1.21±0.22 ng/ml, n=6, p<0.05), but when challenged with TRPA1 agonists in the presence of diazoxide, a significant increase in insulin release was observed (1.64±0.08 ng/ml, n=6, p<0.01), suggesting K_ATP_ channel-mediated depolarization is not required for TRPA1-mediated insulin release (Fig. 6c).

### RNA interference to confirm the role of TRPA1 in pancreatic beta cells

**Figure 7:**
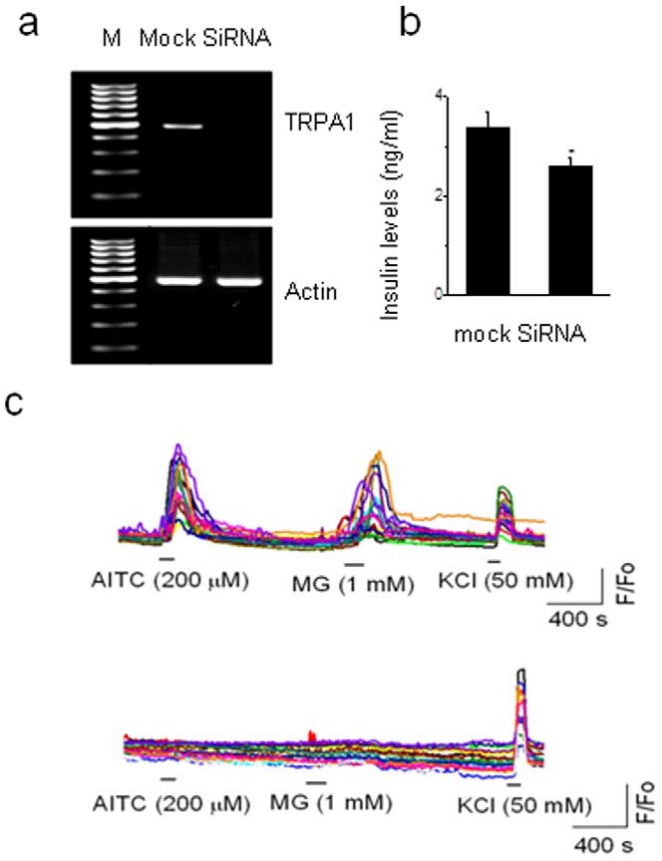
TRPA1-medated insulin release and Ca^2+^ influx following knockdown of TRPA1 by siRNA a. RT-PCR after knockdown of TRPA1 by siRNA in control and mock-transfected cells. b. AITC (200 µM)-induced insulin release is significantly reduced after siRNA knockdown of TRPA1 in RIN cells (n=6, * p<0.05). c. AITC (200 µM) and MG (1 mM) induce increase in intracellular Ca^2+^ levels in mock-transfected RIN cells (upper panel), but not in siRNA treated cells (lower panel). Note that siRNA treated cells responded to KCl (50 mM) similar to that of mock-transfected cells.

In order to further confirm the specificity of involvement of TRPA1 in inducing Ca^2+^ influx and insulin release, the RIN cells were transfected with short interference RNA (siRNA) designed to knockdown TRPA1. Following siRNA transfection of pancreatic beta cells, TRPA1 mRNA was not detected when compared to mock transfected cells, confirming the efficiency of TRPA1 knockdown (Fig. 7a). In the presence of glucose (3 mM) AITC (200 µM)-induced insulin release was significantly diminished (from 3.37±0.31 to 2.60±0.15 ng/ml, n=6, p<0.05) in siRNA transfected cells (Fig. 7b), but not in mock transfected cells. Ca^2+^ influx evoked by TRPA1 agonists, but not by KCl was completely abolished by TRPA1 knockdown in these cells (Fig. 7c). These experiments further confirm TRPA1-mediated insulin release from pancreatic beta cells.

## Discussion

In this study, we have demonstrated the expression of TRPA1 in a pancreatic beta cell line (RIN) and freshly isolated rat pancreatic islets using RT-PCR, Western blot and immunohistochemical techniques. We have further studied the channel function using Ca^2+^ imaging and patch-clamp techniques to show that activation of TRPA1 induces Ca^2+^ influx and membrane depolarization. Finally, we have demonstrated that activation of TRPA1 by exogenous and endogenous agonists causes insulin release.

A number of TRP channels have been shown to be expressed in the pancreas and to be involved in insulin release and with our findings TRPA1 is added to this list. But the relative importance of these TRP channels expressed in the pancreatic beta cells is yet to be clarified. Transcripts of the non-selective cation channels TRPC1 and TRPC4, which mediate Ca^2+^ influx following stimulation of G-protein coupled receptors have been identified [44]–[49]. TRPV1 has been shown to modulate insulin secretion and TRPV2-mediated insulin release is significantly decreased after TRPV2 knockdown [5], [8]. TRPV4 is expressed in pancreatic beta cells and increases intracellular Ca^2+^ when activated by amyloid polypeptide [9]. TRPM2, TRPM3, TRPM4, and TRPM5 channels are associated with Ca^2+^ influx in pancreatic beta cells and involved in insulin release, in particular TRPM3 is directly activated by the neurosteroid pregnenolone sulphate [10]–[15]. It has been shown that TRPA1 is functionally expressed in pancreatic beta cells and proposed to be involved in insulin release [6], [7].

From the results of this study, we propose that TRPA1 is an attractive candidate to be involved in insulin release because it is activated by reactive molecules that are produced during oxidative glycolysis. We have shown that in a pancreatic beta cell line and in freshly isolated rat pancreatic beta cells, application of MG, 4-HNE, PGJ_2_, and H_2_O_2_ is able to increase intracellular Ca^2+^ levels and cause membrane depolarization. We are able to demonstrate that these agonists also cause insulin release. As we are applying endogenous intracellularly produced molecules exogenoulsy, the effective concentrations that are required to activate the channels are not readily determined. In fact, while using MG as an agonist in electrophysiological experiments, we found that exposure of MG to the cytoplasmic surface of the membrane activated TRPA1 with much lower concentrations (EC_50_=590 nM) than the concentrations required to induce a response when applied extracellularly. Although, we have shown that H_2_O_2_ activates TRPA1, it is also implicated in other functions. TRPM2 channels have been shown to be activated by H_2_O_2_ and excessive Ca^2+^ influx may induce beta cell death [45], [50], [51]. K_ATP_ channels activity is also increased by H_2_O_2_ [50]–[52], which leads to membrane hyperpolarization and inhibition of insulin secretion. H_2_O_2_ can also cause ATP depletion, which can trigger Ca^2+^ release from intracellular stores [53]–[55].

It is not clear in what circumstances TRPA1-mediated insulin release comes into play. We are able to show that insulin release induced by exogenous and endogenous ligands can be blocked by TRPA1 antagonists. We have conducted experiments with different concentrations of glucose to determine the role of TRPA1 under normal physiological functions. Following incubation with TRPA1 antagonists, a significant decrease in glucose-induced insulin release was observed suggesting that it has a role in normal physiological conditions. In order to eliminate the possibility of TRPA1-mediated generation of action potentials and the involvement of voltage-gated channels in insulin release, we incubated the cells with voltage-gated Na^+^ channels blocker (TTX) and voltage-gated Ca^2+^ channel blocker (nimodipine). Incubation with these blockers alone caused a decrease in insulin release suggesting their role in the basal insulin release; however, when challenged with TRPA1 agonists, a significant increase in insulin release was observed. These results suggest that TRPA1-mediated insulin release is independent of Ca^2+^ flux through voltage-gated Ca^2+^ channels. In order to eliminate the role of K_ATP_ channel-mediated depolarization, we incubated the cells with the K_ATP_ channel activator, diazoxide. In the presence of diazoxide there was a basal decrease in insulin release, however, when challenged with TRPA1 agonists, a significant increase in insulin release was observed. This is consistent with K_ATP_ channel-mediated hyperpolarization increasing the driving force for Ca^2+^ entry through TRPA1.

In general, insulin secretion is stimulated by glucose, hormones and neurotransmitters. Glucose enters beta cells mainly via the Glut-2 transporter and cytosolic glucose concentration is rapidly adapted to the changes in blood glucose concentration. [52]. The most predominant mechanism of insulin secretion in response to increased blood glucose levels is by the triggering pathway brought about by alteration in ATP/ADP ratio that results in the block of K_ATP_ channel, membrane depolarization, Ca^2+^ influx through voltage-gated Ca^2+^ channels, and exocytosis of insulin containing vesicles [52]. An amplifying pathway due to sensitization of the exocytotic machinery that is independent of K_ATP_ channel activity has been identified [40]–[43], [56], [57]. Insulin release has been studied under conditions, where K_ATP_ channels have been kept open by diazoxide or closed by sulfonylurea. A significant increase in insulin release has been observed with minimal or no change in intracellular Ca^2+^ levels [40]–[42]. The amplifying mechanism strongly depends on metabolism of glucose, however, the signal/s responsible for this phenomenon is/are not yet identified. Incretins such as glucagon-like peptide 1 (GLP-1) induces depolarization, which is independent of K_ATP_ channel inhibitions, it has been suggested that GLP-1 may activate a TRP channels [58]–[60]. However, it is puzzling to learn that during the amplification process, there was no increase in plasmalemma Ca^2+^ concentration [42].

Based on the results presented in this study, we propose a mechanism of insulin release in pancreatic beta cells involving the TRPA1 ion channel. Under normal conditions the TRPA1-mediated pathway may act as a low tone system to cause insulin release to support the trophic functions. However, in hyperglycemic conditions, production of ROS and MG can directly evoke insulin release through this pathway, providing a novel signaling mechanism leading to insulin release in addition to or in concert with block of K_ATP_ channels.

## Supporting Information

Figure S1 **MG-induced dose dependent increase in Ca^2+^ influx. MG (1–1000 µM) evokes concentration-dependent increases in [Ca^2+^]_i_, with an EC_50_ value of 28.6 µM.**
(TIF)

Figure S2 **TRPA1-mediated membrane currents in RIN cells.** **a.** MG- and AITC-induced currents. **b.** MG-induced current is blocked by HC030031. Currents induced by **c**. 4-HNE and **d**. PGJ_2_ are blocked by HC030031 (100 µM).
(TIF)
