# Supplementary figures and images for: Expression of Transient Receptor Potential Ankyrin 1 (TRPA1) and Its Role in Insulin Release from Rat Pancreatic Beta Cells
Source: PLoS One. 2012 May 31;7(5):e38005. doi: 10.1371/journal.pone.0038005 (PMC3365106; doi:10.1371/journal.pone.0038005)

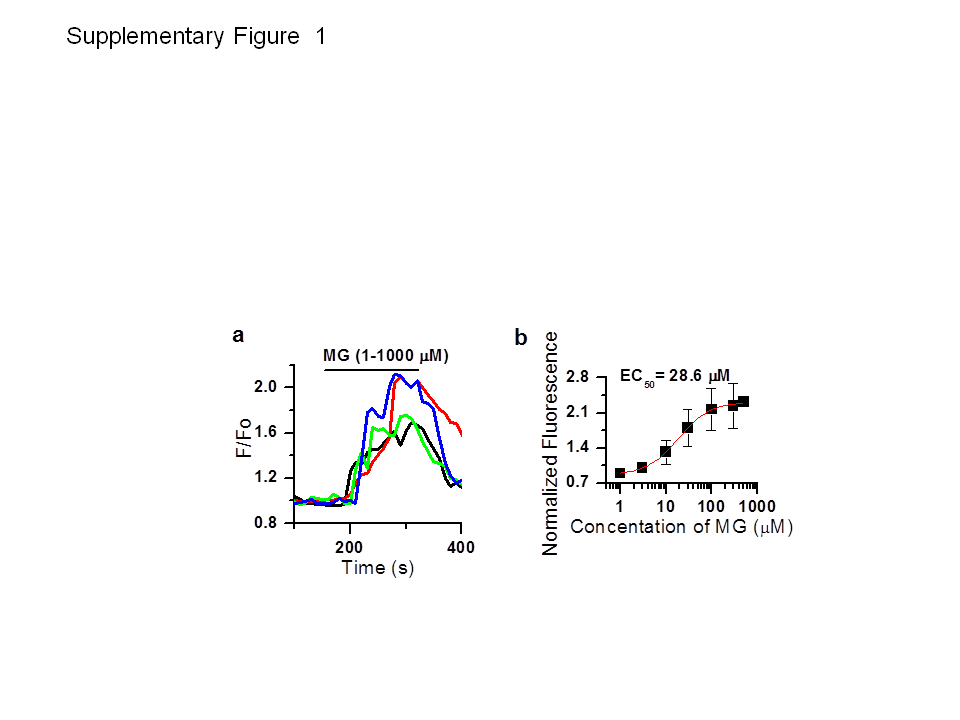

Supplement: Figure S1 — MG-induced dose dependent increase in Ca2+ influx. MG (1–1000 µM) evokes concentration-dependent increases in [Ca2+]i, with an EC50 value of 28.6 µM. (TIF) [file pone.0038005.s001.tif]

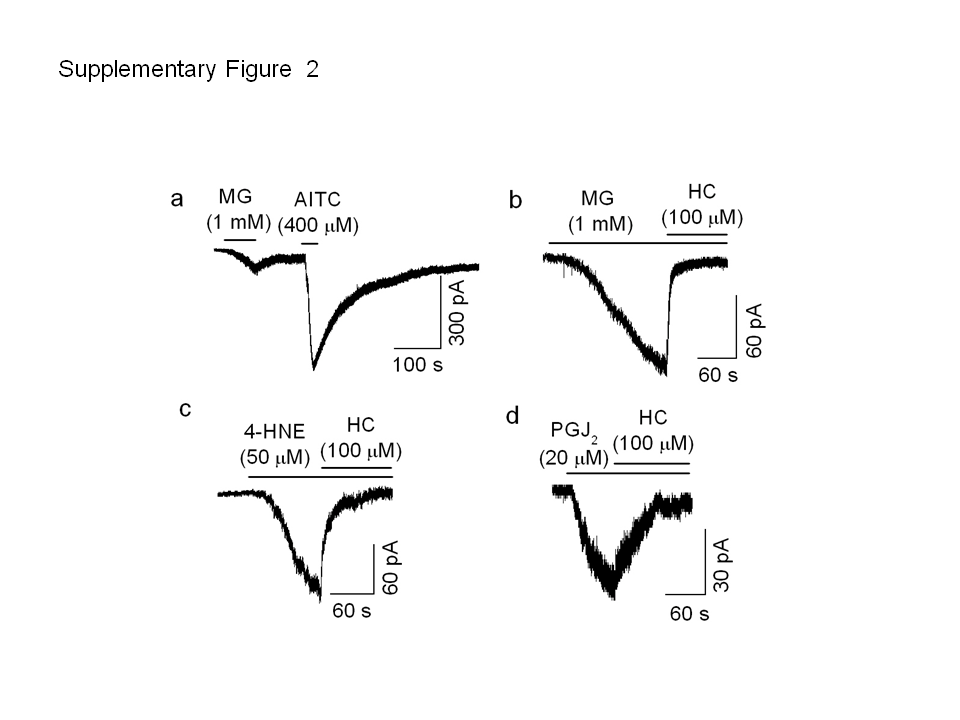

Supplement: Figure S2 — TRPA1-mediated membrane currents in RIN cells. a. MG- and AITC-induced currents. b. MG-induced current is blocked by HC030031. Currents induced by c. 4-HNE and d. PGJ2 are blocked by HC030031 (100 µM). (TIF) [file pone.0038005.s002.tif]
